# On-Chip Polarization Light Microscopy

**DOI:** 10.3390/bios15020079

**Published:** 2025-01-30

**Authors:** Túlio de L. Pedrosa, Renato E. de Araujo, Sebastian Wachsmann-Hogiu

**Affiliations:** 1 Department of Bioengineering, McGill University, Montreal, QC H3A 0G4, Canada; tulio.pedrosa@ufpe.br; 2 Laboratory of Biomedical Optics and Imaging, Federal University of Pernambuco, Recife 50740-550, Brazil; renato.earaujo@ufpe.br

**Keywords:** CMOS image sensors, quantitative polarization light microscopy, point-of-care/need applications

## Abstract

Polarization light microscopy (PLM) enables detailed examination of birefringent materials and reveals unique features that cannot be observed under non-polarized light. Implementation of this technique for quantitative PLM (QPLM) assessment of samples is challenging and requires specialized components and equipment. Here, we demonstrate QPLM on a semiconductor imaging chip that is suitable for point-of-care/need applications. A white LED illumination was used with crossed polarizers and a full wave plate to perform on-chip, non-contact-mode QPLM. Polarization complexity is probed by assessing the multispectral phase shift experienced by white light through the distinct optical paths of the sample. This platform can achieve micrometer-scale spatial resolution with a Field of View determined by the size of the semiconductor sensor. Visualization of a biological sample (*Euglena gracilis*) was demonstrated, as well as the detection of Monosodium Urate crystals, where the presence of negative birefringence of crystals in synovial fluid is important for the diagnosis of gout.

## 1. Introduction

Polarization light microscopy (PLM) is a technique that enables detailed examination of the internal structures of birefringent materials and reveals unique features that are often invisible under non-polarized light. While this technique has been extensively employed for the assessment of birefringent samples, quantitative assessment of PLM presents a more complex challenge. Nevertheless, steady advances made in polarization detection in recent years, such as improvements in imaging techniques, automation and integration with other technologies, have enabled researchers to use PLM to better probe structural details of anisotropic specimens at the microscopic level.

Polarimetric measurements are a very significant source of information in biomedical imaging. For instance, metasurface-assisted differential interference contrast microscopy (DIC) was demonstrated for real-time label-free cellular imaging in the observation of unstained breast cancer tissues, exhibiting sharper contrast at cell boundaries when compared to regular DIC [1]. Moreover, the combination of PLM with modulation techniques such as optical heterodyne interferometry and polarized structured illumination microscopy (pSIM) allows for the detection of individual metal nanoparticles [2] and enables super-resolution imaging, explored in the detection of biological filamentous systems such as cytoskeleton networks and in imaging the dynamics of protein-labeled microtubules in live cells [3,4]. Moreover, the combination of PLM with other microscopy techniques, such as fluorescence microscopy, offers high levels of sensitivity for disease diagnosis such as for cancer [5], in which complementary information provided by the multimodal approach has been applied to assess the severity of the cancer invasion, beyond simple tumor detection. Furthermore, optical diffraction tomography has also been used in conjunction to PLM to successfully identify birefringent Monosodium Urate (MSU) crystals by imaging unlabeled intracellular components and measuring the three-dimensional refractive index of synovial leukocytes of patients with gout [6], while polarimetric measurements have been used to discriminate between normal and abnormal cells in pancreatic tissues [7]. The addition of polarization masks and metasurfaces to high-resolution image sensors is a promising prospect for real-time full-Stokes polarimetric measurements of biological samples [8]. Equipped with machine learning algorithms, image analysis can assist in the identification of patterns in complex datasets, which has already been used to improve the efficiency of polarimetric characterization of cancer by aiding in the classification of non-melanoma skin cancer tissues in mice [9] and by accomplishing virtual birefringence imaging and virtual tissue staining in label-free tissue slides of amyloid deposits of cardiac patients [10]. Additionally, quantitative birefringence retardance and orientation information of MSU has been demonstrated in a lensless holographic imaging system using machine learning [11]. However, machine learning algorithms rely heavily on the data they are trained on. Obtaining accurate and reliable data can be challenging, especially for healthcare in resource-poor environments.

In this framework, the development of portable, lensless microscopy systems enables real-time analysis of samples in situ, facilitating immediate data collection and interpretation. While lens-based imaging requires costly and bulky optical components, lensless imaging offers high-resolution capability limited only by the pixel size of the sensor (as small as 0.7 μm) with an FoV that typically covers the entire sensor area. Therefore, lensless methodologies enable enhanced portability and cost-effectiveness compared with their lens-based counterparts [12]. Lensless on-chip imaging is categorized into non-contact mode, in which the sample is at a distance greater than 10 μm from the sensor, and contact mode, where the sample is in direct contact with the active area of the sensor. Both cases have been demonstrated for different microscopy modalities, including dark field [13], fluorescence [14], electrochemiluminescence [15], bioluminescence [16] and polarization [11,17,18]. While several lensless on-chip demonstrations for PLM have been published, those experimental designs are relatively complex as they require intricate illumination schemes such as partially coherent light sources, optical masks and programmable light switching.

Successful techniques such as Polychromatic Polarization Microscopy (PPM) have been developed to detect sample birefringence. Although PPM has been employed to detect MSU crystals in joint fluid aspirates [19], its use relies on a conventional polarization microscope used in clinical settings. Given the advantages of lensless on-chip platforms for the development of compact imaging devices, we report here a compact optical system for non-contact-mode quantitative PLM. This system utilizes crossed polarizers and a full wave plate to probe polarization complexity by assessing the multispectral phase shift experienced by white light through the distinct optical paths of a birefringent sample. This implementation provides a low-cost alternative to lens-based systems and is advantageous for point-of-care/need applications that require simplicity and scalability in resource-poor environments.

## 2. Materials and Methods

### 2.1. Optical Componentes

Epoxy-encased white light LED was used as the illumination source. The visible spectrum of the LED source was obtained using the ST VIS Microspectrometer (Ocean Optics, Orlando, FL, USA). A 50 μm diameter stainless steel pinhole was acquired from Thorlabs (Newton, NJ, USA). An optical lens (PMMA) of 18 mm diameter, 6 mm thickness and f = 50 mm was used. A sheet of linear polarizer film (300 × 200 mm) was acquired from K&F Concept (extinction rate ≈ 3.8%, polarization efficiency ≈ 92%) (Shenzhen, China). The full waveplate film (WP560) was acquired from Edmund Optics (Barrington, NJ, USA). The CMOS sensors (model OV5647, 3.68 × 2.76 mm, 2592 × 1944 pixels and pixel size of 1.4 µm) used in this report were purchased from OmniVision Technologies.

### 2.2. Samples

The performance of the on-chip polarization microscopy system was evaluated using five different sample types: Mica plate, Ascorbic Acid Crystals, MSU crystals, Euglena cells and Polystyrene beads.

Sodium hydroxide (>99%) and ascorbic acid (>99%) were purchased from Sigma-Aldrich. Uric acid (UB0978, >99%) was purchased from Bio Basic Inc. (Markham, ON, Canada). Polystyrene beads with 1.6 µm diameter were purchased from Corpuscular, Inc. (Cold Spring, NY, USA), and diluted in Milli-Q^®^ water (1: 10000 *v*/*v* dilution) before use.

*Euglena gracilis* was purchased from Carolina Biological Supply Company (Burlington, NC, USA) and grown in liquid Provasoli’s enriched seawater (PES) medium at room temperature. *Euglena gracilis* was used in experiments by pipetting a few microliters of the microorganism from its liquid culture onto the analyzer (LP 2).

#### 2.2.1. MSU Crystal Synthesis

The synthesis of supersaturated solution containing MSU crystals was based on the procedures described by Liu et al. [20]. Here, 2.44 mL of 1 M Sodium hydroxide was dissolved in 80 mL of Milli-Q^®^ water and heated to 100 °C, after which 400 mg of uric acid was added. The temperature was then lowered to 60 °C and kept for 5 h. Finally, the solution was allowed to cool down to room temperature and was left stirring overnight. The sample was conserved in supersaturated solution and stored at room temperature in a quiet environment.

A total of 5 μL of supersaturated solutions of MSU was drop-casted on top of a coverslip and left to dry in open air, at room temperature. The precipitated crystals were then analyzed using Bright Field microscopy (WITec Alpha300R Confocal Raman Microscopy system) equipped with a 50× objective (NA 0.8, WD 0.58 mm) to evaluate the needle-like structures formed during the crystallization process [21].

#### 2.2.2. Ascorbic Acid Crystal Synthesis

To produce the ascorbic acid (AA) sample, 26.4 g of AA powder was dissolved in 80 mL of Milli-Q^®^ water and heated close to the boiling point. While stirring, small amounts of AA were added to the solution in incremental steps until the saturation point was reached. The saturation point was determined by visual inspection.

### 2.3. Fabrication of the Lensless Cross-Polarization Device

**Figure 1:**
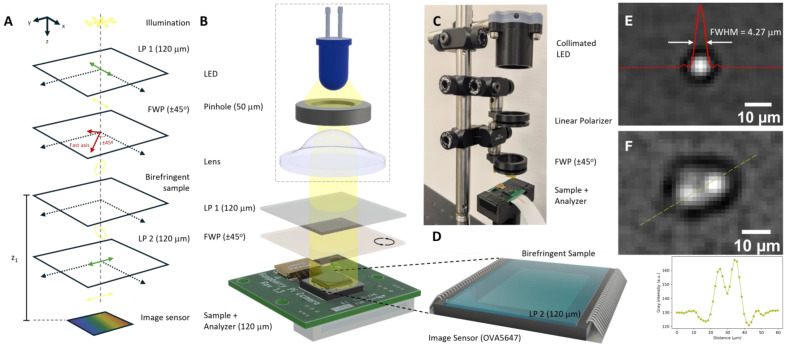
On-chip lensless cross-polarization platform. (**A**). Schematic diagram of the transformations imposed to the polarization of light as it propagates down to the image sensor. LP 1—linear polarizer, FWP—full waveplate and LP 2—linear polarizer (cross-polarizer); (**B**). exploded view of the main elements of the device; (**C**). experimental setup implementation; (**D**). close-up of the polarization analyzer module (image sensor + cross-polarizer); and lateral resolution test using (**E**). one bead; and (**F**) two adjacent polystyrene beads. (outset: intensity profile for the two beads along the yellow dashed line).

The cross-polarization device comprises four main parts, assembled with the help of 3D-printed parts, as shown in Figure 1C: (i) a collimated LED source; (ii) a linear polarizer; (iii) a 1λ retarder (full waveplate, FWP); and (iv) an analyzer module. The 3D-printed parts provide the flexibility needed for adjustments during the measurement procedure. Figure 1B depicts the system in greater detail: the non-polarized illumination source (white LED, 5 mm, 15 mW, 7.5° half viewing angle) is filtered by a 50 µm pinhole, after which the resulting point source illumination is collected by a lens mounted in a threaded tube. The distance from the lens to the pinhole was adjusted until a collimated beam was produced. The collimated beam of light was then polarized (LP 1, 120 µm thickness) and moved through the FWP (150 µm thickness) positioned at ±45°, both of which were placed in manual rotating mounts for angle control. The cross-polarizer (LP 2, 120 µm thickness) and the image sensor formed the polarization analyzer (Figure 1D), used to discriminate the polarization of light that reaches the sensor.

### 2.4. Polarization Measurements

The experimental setup comprises of a non-polarized illumination source, a set of cross-polarizers and a full waveplate. The illumination source is collimated and then polarized by LP 1. As soon as LP 1 is introduced, only one polarization interacts with the birefringent sample. The polarized light is split into two components (ordinary and extraordinary), which introduces a difference in the number of wavelengths within the sample’s pathlength. The recombination of light after emerging from the sample results in a different phase shift and polarization (since the effective polarization vector is rotated). This effect occurs for all wavelengths. The FWP is used to add or subtract effective retardance from the sample, depending on its orientation in relation to the polarization of incident light (±45°). Then, the polarization analyzer LP 2 reveals a colorful interference pattern that indicates the amount of birefringence of the sample. The image sensor registers this pattern as an image for future data processing. By rotating the full waveplate around ±45°, different interference color patterns arise. In this sense, the colorful images obtained with our setup can be explored to extract quantitative data about the birefringence of the samples.

#### 2.4.1. Colorimetric Evaluation

Human vision takes a complex spectral distribution (color) that can be represented by the tristimulus values (X, Y, Z), defined by the International Commission on Illumination (CIE) as [22].

(1) $$X=\int_{\lambda_{i}}^{\lambda_{f}}I\left(\lambda \right)\bar{x}\left(\lambda \right)d\lambda ,$$

(2) $$Y=\int_{\lambda_{i}}^{\lambda_{f}}I\left(\lambda \right)\bar{y}\left(\lambda \right)d\lambda \;\mathrm{and}$$

(3) $$Z=\int_{\lambda_{i}}^{\lambda_{f}}I\left(\lambda \right)\bar{z}\left(\lambda \right)d\lambda .$$

Here, *I(λ)* is the spectral power distribution of the light source and $\bar{x}\left(\lambda \right),$ $\bar{y}\left(\lambda \right)$ and $\bar{z}\left(\lambda \right)$ are the standard color matching functions [21]. The integrations were performed over the visible spectrum, with initial and final wavelengths of $\lambda_{i}=380$ nm and $\lambda_{f}=780$ nm, respectively.

The human perception of color change also depends on the intensity of the observed light. Therefore, the use of the CIELAB color space is preferred to represent visual differences in color. Human perception of color differences can be quantified by the CIEDE2000 formula within the CIELAB space [23]. Color cameras such as the CMOS sensor used in this work exploit a Bayer filter coupled to processing algorithms to reproduce colors that match the perception of the human eye. Therefore, understanding the perceptual differences between colors is essential. In this context, the perceived difference in color (ΔE) calculated using the CIEDE2000 formula serves as a crucial metric in birefringence estimation by comparing color distances in the imaged color interference pattern that arises from samples under cross-polarization. The PLM colorimetric evaluation considers that light is transmitted through the platform/sample, and no attenuation is explicitly considered.

#### 2.4.2. Jones Matrix Formalism

By applying the Jones matrix formalism to account for polarization rotation for each spectral component of the illumination source, color images can be used to extract quantitative data from birefringent samples in a process that transforms the emerging interference colors into phase differences, resulting in phase images. Our approach calculates polarization rotations for each wavelength of the spectral power distribution of the chosen illumination source (white LED) as it propagates through the optical path. The effective interference color is then calculated, based on Equations (1)–(3), exploring the resulting spectral power distribution that reaches the image sensor. Waveplates and polarizers were modeled analytically using Jones formalism. Therefore, the polarization rotation detected at the CMOS sensor for a given wavelength, $J_{T_{xy}}\left(\lambda \right)$, is [24]

(4) $$J_{T_{xy}}\left(\lambda \right)=M_{y-pol}\left(\lambda \right)M_{sample}\left(\lambda \right)M_{fwp}\left(\pm \frac{\pi }{4},\lambda \right)J_{xy}(\lambda )$$

in which $J_{xy}(\lambda )$ represents the x-polarized (horizontal) light and $M_{y-pol}\left(\lambda \right)$ is the Jones matrix for the y-axis (vertical) polarizer for a given wavelength. The spectral distribution of the light source must be considered, as it directly affects the resulting colors. The full waveplate (FWP), $M_{fwp}\left(\pm \frac{\pi }{4},\lambda \right)$, and the birefringent sample, $M_{sample}\left(\lambda \right)$, are modeled as linear phase retarders with wavelength dependence, as follows [24]:

(5) $$M_{fwp}\left(\pm \frac{\pi }{4},\lambda \right)=R\left(\pm \frac{\pi }{4}\right)\left[\begin{array}{cc} e^{j\phi_{0}(\lambda )/2} & 0\\ 0 & e^{-j\phi_{0}(\lambda )/2} \end{array}\right]\;\mathrm{and}$$

(6) $$M_{sample}\left(\lambda \right)=R\left(\frac{\pi }{4}\right)\left[\begin{array}{cc} e^{j\phi (\lambda )/2} & 0\\ 0 & e^{-j\phi (\lambda )/2} \end{array}\right].$$

Here, $R\left(\pm \pi /4\right)$ is the rotation matrix at ±45°, $\phi_{0}(\lambda )$ is the FWP phase shift and ϕ(λ) is the phase shift introduced by sample birefringence. Moreover, to derive the color change introduced by the sample exploring the effective retardance, the optic axis of the sample is positioned 45° relative to the axis of the *xy*-plane, as indicated by the rotation matrix in Equation (6). For each wavelength, the spectral power distribution, *I(λ)*, is retrieved taking $\left(J_{T_{xy}}\cdot J_{T_{xy}}^{*}\right)$. Therefore, for each phase shift, the resulting spectral power distribution is computed into a specific color and stored as a reference for the future retrieval of phase shift from a sample placed in the experimental setup.

#### 2.4.3. Phase-Retrieval Algorithm

In the PLM system, as light propagates through the sample, a spatial phase distribution emerges, leading to a color map of the sample on the image sensor. Knowing the illumination spectrum and waveplate rotation of the imaging system, the phase shift introduced by the birefringent sample can be extracted from the interference colors, using the CIE’s color matching functions (Equations (1)–(3)) and Jones matrix formalisms (Equations (4)–(6)). In other words, the quantification of color changes in the PLM image, based on the CIEDE2000 (ΔE) metric, can be translated into phase shifts, and vice versa.

However, the periodic nature of phase shift promotes color similarity along different interference orders. Therefore, for each pixel of the image sensor, its color is compared with the computed reference colors using the CIEDE2000 formula. Pixel’s phase shift value is determined by color similarity, i.e., minimizing the ΔE metric. Furthermore, as white light is employed, the average phase difference in the visible range, $\langle \Delta \phi \rangle$, must be considered for the extraction of quantitative data from a real sample.

## 3. Results and Discussion

**Figure 2:**
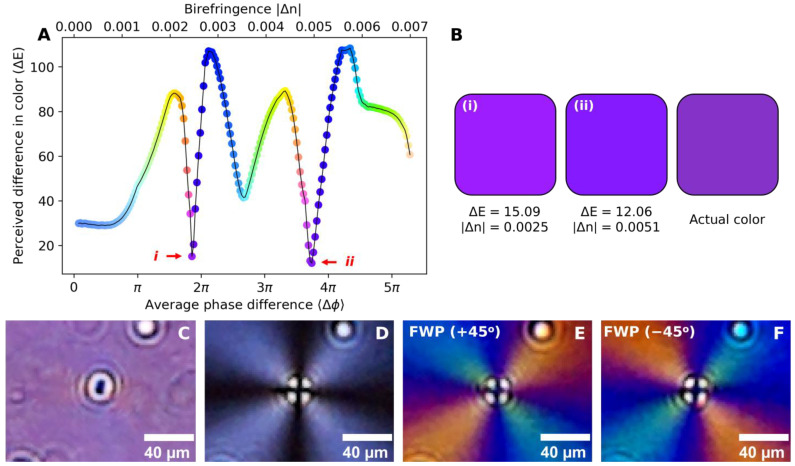
(**A**). Color difference values (CIEDE2000) observed on PLM images considering different averaged phase shifts. The circles show the color produced for a given averaged phase shift; (**B**). color of mica sample on the PLM system, considering (i) $\langle \Delta \phi \rangle =1.89\pi$ and (ii) $\langle \Delta \phi \rangle =3.85\pi$; (**C**). high birefringence center in AA without cross-polarization; (**D**). high birefringence center in AA under cross-polarization; (**E**). high birefringence center in AA under cross-polarization with FWP positioned at +45°; and (**F**). high birefringence center in AA under cross-polarization with FWP positioned at −45°.

Phase-retrieval methodology allows for the association of the observed color of a PLM image with the induced average phase difference due to light propagation through the birefringent sample. Figure 2A indicates the calculated color difference values (CIEDE2000), raised from the induced phase shift, considering a mica sample placed in the on-chip PLM system, with its optical axis positioned 45° relative to the axis of the *xy*-plane. The correlation between interference colors, sample thickness and birefringence are well known and documented by the Michel-Lévy interference color chart [25]. For a single wavelength, phase shift is given by

(7) $$\Delta \phi =\frac{2\pi d\;\Delta n\left(\lambda \right)}{\lambda },$$

where *d* is the sample thickness. As a broad-spectrum light is employed on the PLM system, we apply the Average Function Value integral to the phase shift equation:

(8) $$\langle \Delta \phi \rangle =\frac{2\pi d\;}{\left(\lambda_{f}-\lambda_{i}\right)}\int_{\lambda_{i}}^{\lambda_{f}}\frac{\Delta n\left(\lambda \right)}{\lambda }d\lambda .$$

As a first approximation, Δn is taken out of the integral by assuming it is constant; therefore, the sample birefringence, Δ*n*, is given by

(9) $$\Delta n=\frac{\left(\lambda_{f}-\lambda_{i}\right)\langle \Delta \phi \rangle \;}{2\pi d\ln\left(\lambda_{f}/\lambda_{i}\right)},$$

By identifying the sample color in the imaging system and knowing the sample thickness, it is possible to determine the value of Δn. For example, considering the images of the leftmost and middle boxes in Figure 2B, it can be seen from Figure 2A that the respective colors correspond to phase variations of 1.89π and 3.85π. These colors represent the regions highlighted by red arrows in Figure 2A.

To illustrate the birefringence quantification methodology, a flat piece of birefringent mica of known thickness (d = 210 µm) was introduced in the setup, replacing the FWP (see Figure 1B). The mica sample was adjusted to have its optical axis 45° to the *xy*-plane axis. Thus, the on-chip PLM produced an image containing only one color, uniform across the FoV and illustrated by the rightmost box of Figure 2B. This color was then compared against each color recorded using the steps described in Section 2.4.2 and Section 2.4.3, resulting in Figure 2A. Furthermore, the minimization criterion of color similarity was applied to the possible results and ΔE was minimized. Therefore, for minimum ΔE values, the calculated birefringence obtained by this method was estimated as 0.0051 (ii), a close match to the actual mica birefringence (0.0054) [26].

Figure 2C–F depict one of such high birefringence centers of the AA sample, imaged under our on-chip cross-polarization device for different polarization and FWP configurations. For that, 5 µL of supersaturated solution of AA was drop-casted onto a coverslip, placed on top of the polarization analyzer and left to dry in open air, forming crystal centers surrounded by a thin crystal sheet with radially aligned optic axes. All figures depict the same crystal center. Figure 2C,D depict, respectively, the birefringence center for LP1 removed and LP1 reinserted in the optical path (sample under cross-polarized light). Figure 2E,F emphasize the interference colors that appear by inserting the FWP at +45° (Figure 2E) and −45° (Figure 2F) in the optical path. Additionally, Figure 2D–F show a Maltese cross, an interference pattern that arises from uniaxial crystals under cross-polarization [27]. Its presence is an indicative of sample birefringence and allows for easy identification of specimens found in nature, where it has been used to identify starch grains [28] and fatty molecules [29].

**Figure 3:**
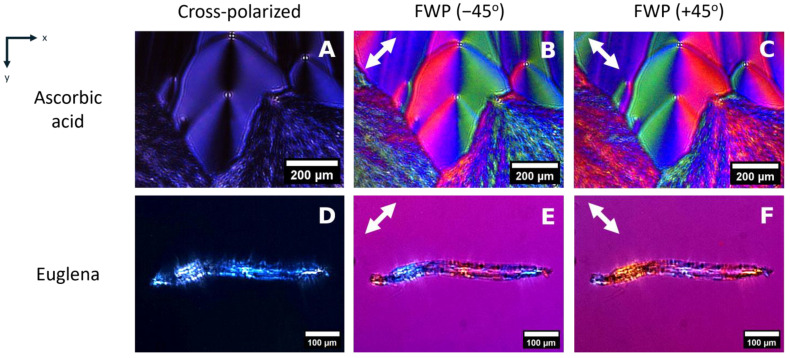
Birefringent samples in the on-chip cross-polarized device: (**A**). AA sample under cross-polarization; (**B**). under cross-polarization with FWP positioned at −45°; and (**C**). under cross-polarization with FWP positioned at +45°. (**D**). *Euglena gracilis* sample under cross-polarization; (**E**). *Euglena gracilis* sample under cross-polarization with FWP positioned at −45°; and (**F**). *Euglena gracilis* sample under cross-polarization with FWP positioned at +45°. The white arrows depict the FWP rotation in the *xy*-plane.

In Figure 3, the effect of the full waveplate in the production of interference colors in birefringent samples is shown. The cross-polarized images of Figure 3A,D demonstrate the sample birefringence in the absence of the FWP, as light depolarized by the sample is allowed to reach the sensor. A crystalized sample of AA with centrosymmetric patterns around the crystal centers is shown in Figure 3A–C, in which the total FoV of the device is demonstrated. The introduction of the FWP in the optical path contributes to the overall phase shift. Rotating the FWP by ±45° adds (subtracts) phase shift to the sample and induces changes in the interference colors by producing higher or lower order interference patterns. By changing FWP orientation, the local birefringence of the sample is revealed, highlighted by the color “mirroring” that ensues. This phenomenon is material-dependent and is used for sample identification in fields such as geology [30] and rheumatology [31]. Here, the addition and subtraction of phase shift is also visualized in living organisms (*Euglena gracilis*) (Figure 3D–F). The dependence of the image colors on the FWP angle is also depicted in Figure 3E,F. Without the FWP, the interference wavelength is too small, and the colors appear washed (Figure 3A,D). Phase shift is introduced by adding the FWP through the increase in interference order. Positioning the FWP at ±45° produces slight changes in the interference colors and enables sample identification (Figure 3B,C,E,F).

In a clinical setting, detecting MSU crystals through polarized light microscopy is regarded as the gold standard for diagnosing gout. Uric acid, a byproduct of purine metabolism, is typically excreted naturally. MSU crystals, one of the most prevalent salts derived from uric acid ions, can form in the renal tubules, ureters and synovial or soft tissues. The precipitation of these crystals in the synovial fluid triggers an inflammatory response that results in intense pain. Therefore, the presence of MSU in synovial fluid indicates the presence of gout [19,32].

**Figure 4:**
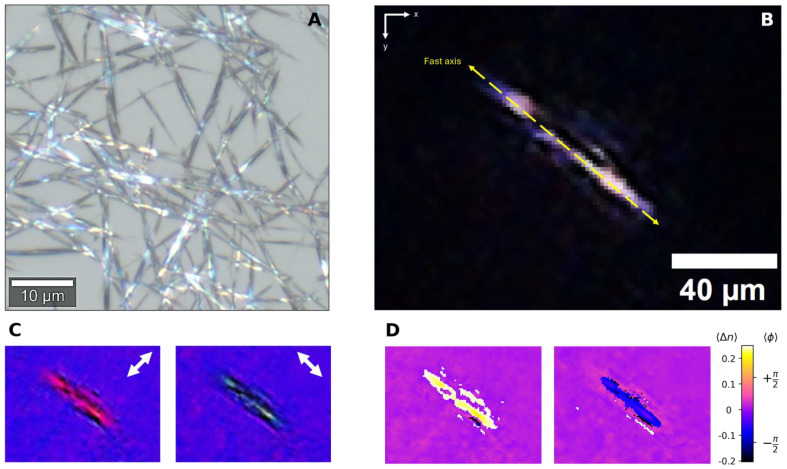
(**A**). Bright Field image of the MSU crystals after synthesis; (**B**). cross-polarization image of a single MSU crystal in the PLM system, without FWP. (**C**). Same MSU crystal under cross-polarization with FWP at ±45°. These are the real colors imaged by the CMOS sensor used for (**D**). Birefringence maps reconstructed for the sample. The white arrows depict the FWP rotation in the *xy*-plane.

Synthesized MSU crystals were used to mimic the optical response of gout in synovial fluid. Figure 4A shows a Bright Field microscopy image (50× objective-NA 0.8, WD 0.58 mm) of the synthesized MSU crystals, with their typical long needle-shape and crystal thickness of 1.1 µm. Due to their symmetry, the typical thickness of the MSU crystals was estimated measuring the short axis length of the crystal using WITec Alpha300R Confocal Raman Microscopy system software. The MSU sample was drop-casted in the lensless setup and imaged under cross-polarization. Figure 4B illustrates the high contrast achieved between birefringent MSU crystal and the non-birefringent background, typical of cross-polarized experiments. Such crystals show strong negative birefringence under polarized light [17]. Their long axis is the “fast” axis (yellow dashed line). The apparent size of the crystal is misleading, due to the separation between the sample and CMOS sensor. The FWP was used to produce additional retardance and enhance interference colors, as depicted in Figure 4C. If the long axis of a gout crystal is aligned with the slow axis of the FWP, the resulting phase difference should result in a yellow interference color. Conversely, when the long axis of a gout crystal is aligned with the fast axis of the FWP, the phase difference results in a blue color. In Figure 4D, the interference colors are processed, and a phase image is obtained for the −45° (left) and the +45° cases (right). The colors are virtual and were chosen to match the expected MSU colors in a clinical setting.

For the proposed cross-polarization setup, image resolution is highly limited by the distance between the sample and the CMOS sensor and the pixel size. Therefore, the thickness of LP2 in the analyzer module is a constraint of utmost importance to our limits of detection. Consequently, spreading of the rays after passing the object produces an image larger than the actual sample.

To evaluate the optical resolution of the system, 2 µL of 1.6 µm polystyrene (PS) beads was drop-casted onto a coverslip, placed on top of the polarization analyzer. After drying at room temperature, the PS beads were imaged, as depicted in Figure 1E. The apparent PS bead size was obtained fitting a *sinc* function to the intensity profile diffracted by the bead, resulting in an FWHM of 4.27 µm. The apparent larger size of the PS bead arises from the point spread function. Reducing sample distance to the CMOS sensor should reduce the measured bead diameter. Figure 1F depicts two resolved (within the Rayleigh criteria) PS beads. The outset represents the intensity profile along the yellow dashed line. Furthermore, the use of CMOS sensors of smaller pixel size would result in more detailed images, without resorting to signal processing techniques and intricate illumination schemes.

## 4. Conclusions

On-chip PLM was implemented exploring a simple cross-polarization platform with a rotating full waveplate for the imaging of birefringent samples. A methodology for quantitative color analysis was proposed through the modeling of birefringence using Jones formalism. The broadband character of the illumination source combined with the mathematical modeling of the interference colors was investigated for phase reconstruction. Our compact platform has the potential to achieve micrometer-scale spatial resolution with a Field of View determined by the size of the semiconductor sensor (in our case, 3.68 mm × 2.76 mm). Visualization of a biological sample (*Euglena gracilis*) was demonstrated, where the correlation between interference colors, sample thickness and birefringence are well known and documented by the Michel-Lévy interference color chart. Moreover, a demonstration of this technique for the detection of MSU crystals was shown, where the presence of negative birefringence of crystals in synovial fluid is important for the diagnosis of gout. Although the proposed method enables the quantitative characterization of birefringent materials, its application is subject to certain limitations, such as the need for precise control of sample orientation and accurate knowledge of the sample’s dimensions. Overall, this technique can be further miniaturized and built into a portable, point-of-care/need device that can be applied to a variety of biological and non-biological samples.

## Data Availability

The data supporting the findings of this study are available within the article. Additional data can be obtained from the corresponding author upon reasonable request.

## References

[1] Wang X., Wang H., Wang J., Liu X., Hao H., Tan Y.S., Zhang Y., Zhang H., Ding X., Zhao W. (2023). Single-Shot Isotropic Differential Interference Contrast Microscopy. Nat. Commun..

[2] Hong X., Van Dijk E.M.P.H., Hall S.R., Götte J.B., Van Hulst N.F., Gersen H. (2011). Background-Free Detection of Single 5 nm Nanoparticles through Interferometric Cross-Polarization Microscopy. Nano Lett..

[3] Zhanghao K., Chen X., Liu W., Li M., Liu Y., Wang Y., Luo S., Wang X., Shan C., Xie H. (2019). Super-Resolution Imaging of Fluorescent Dipoles via Polarized Structured Illumination Microscopy. Nat. Commun..

[4] Guan M., Wang M., Zhanghao K., Zhang X., Li M., Liu W., Niu J., Yang X., Chen L., Jing Z. (2022). Polarization Modulation with Optical Lock-in Detection Reveals Universal Fluorescence Anisotropy of Subcellular Structures in Live Cells. Light Sci. Appl..

[5] Liu Y., York T., Akers W., Sudlow G., Gruev V., Achilefu S. (2012). Complementary Fluorescence-Polarization Microscopy Using Division-of-Focal-Plane Polarization Imaging Sensor. J. Biomed. Opt..

[6] Park S., Lee L.E., Kim H., Kim J.E., Lee S.J., Yoon S., Shin S., Kang H., Park Y., Song J.J. (2021). Detection of Intracellular Monosodium Urate Crystals in Gout Synovial Fluid Using Optical Diffraction Tomography. Sci. Rep..

[7] Sampaio P., Lopez-Antuña M., Storni F., Wicht J., Sökeland G., Wartenberg M., Márquez-Neila P., Candinas D., Demory B.-O., Perren A. (2023). Müller Matrix Polarimetry for Pancreatic Tissue Characterization. Sci. Rep..

[8] Zuo J., Bai J., Choi S., Basiri A., Chen X., Wang C., Yao Y. (2023). Chip-Integrated Metasurface Full-Stokes Polarimetric Imaging Sensor. Light Sci. Appl..

[9] Pham T.-T.-H., Luu T.-N., Nguyen T.-V., Huynh N.-T., Phan Q.-H., Le T.-H. (2023). Polarimetric Imaging Combining Optical Parameters for Classification of Mice Non-Melanoma Skin Cancer Tissue Using Machine Learning. Heliyon.

[10] Yang X., Bai B., Zhang Y., Aydin M., Li Y., Selcuk S.Y., Casteleiro Costa P., Guo Z., Fishbein G.A., Atlan K. (2024). Virtual Birefringence Imaging and Histological Staining of Amyloid Deposits in Label-Free Tissue Using Autofluorescence Microscopy and Deep Learning. Nat. Commun..

[11] Liu T., De Haan K., Bai B., Rivenson Y., Luo Y., Wang H., Karalli D., Fu H., Zhang Y., Fitzgerald J. (2020). Deep Learning-Based Holographic Polarization Microscopy. ACS Photonics.

[12] Hu X., Abbasi R., Wachsmann-Hogiu S. (2023). Microfluidics on Lensless, Semiconductor Optical Image Sensors: Challenges and Opportunities for Democratization of Biosensing at the Micro-and Nano-Scale. Nanophotonics.

[13] Imanbekova M., Perumal A.S., Kheireddine S., Nicolau D.V., Wachsmann-Hogiu S. (2020). Lensless, Reflection-Based Dark-Field Microscopy (RDFM) on a CMOS Chip. Biomed. Opt. Express.

[14] Kuo G., Linda Liu F., Grossrubatscher I., Ng R., Waller L. (2020). On-Chip Fluorescence Microscopy with a Random Microlens Diffuser. Opt. Express.

[15] Abbasi R., Wachsmann-Hogiu S. (2024). Optimization and Miniaturization of SE-ECL for Potential-Resolved, Multi-Color, Multi-Analyte Detection. Biosens. Bioelectron..

[16] Abbasi R., Imanbekova M., Wachsmann-Hogiu S. (2024). On-Chip Bioluminescence Biosensor for the Detection of Microbial Surface Contamination. Biosens. Bioelectron..

[17] Zhang Y., Lee S.Y.C., Zhang Y., Furst D., Fitzgerald J., Ozcan A. (2016). Wide-Field Imaging of Birefringent Synovial Fluid Crystals Using Lens-Free Polarized Microscopy for Gout Diagnosis. Sci. Rep..

[18] Kim J., Song S., Kim H., Kim B., Park M., Oh S.J., Kim D., Cense B., Huh Y.M., Lee J.Y. (2023). Ptychographic Lens-Less Birefringence Microscopy Using a Mask-Modulated Polarization Image Sensor. Sci. Rep..

[19] Jum’ah H., Shribak M., Keikhosravi A., Li B., Liu Y., Obaidat D., Eliceiri K.W., Loeffler A., Ayub S. (2023). Detection of Crystals in Joint Fluid Aspirates with Polychromatic Polarisation Microscopy. Ann. Rheum. Dis..

[20] Liu E., Dalbeth N., Pool B., Ramirez Cazares A., Ranganath V.K., FitzGerald J.D. (2023). Ultrasound Findings of Monosodium Urate Aggregates in Patients with Gout. Gout Urate Cryst. Depos. Dis..

[21] Martillo M.A., Nazzal L., Crittenden D.B. (2014). The Crystallization of Monosodium Urate. Curr. Rheumatol. Rep..

[22] Fairman H.S., Brill M.H., Hemmendinger H. (1997). How the CIE 1931 Color-Matching Functions Were Derived from Wright-Guild Data. Color Res. Appl..

[23] Luo M.R., Cui G., Rigg B. (2001). The Development of the CIE 2000 Colour-Difference Formula: CIEDE2000. Color Res. Appl..

[24] Collett E. The Jones Matrix Calculus. Field Guide to Polarization.

[25] Sørensen B.E. (2013). A Revised Michel-Lévy Interference Colour Chart Based on First-Principles Calculations. Eur. J. Mineral..

[26] El-Bahrawi M.S., Nagib N.N., Khodier S.A., Sidki H.M. (1998). Birefringence of Muscovite Mica. Opt. Laser Technol..

[27] Pečar M., Čepič M. (2015). Conoscopic Figure: A Complex Consequence of a Not so Simple Phenomenon. Eur. J. Phys..

[28] Ghosh S., Roy A. (2023). Optical Anisotropy and Dimple Formation on Films Formed after Drying of Gelatinized Starch Solution Droplets. ACS Omega.

[29] Moon J., Seo K., Kang H. (2021). Vertical Alignment of Liquid Crystals on Comb-Like Renewable Chavicol-Modified Polystyrene. Polymers.

[30] Cesare B., Campomenosi N., Shribak M. (2022). Polychromatic Polarization: Boosting the Capabilities of the Good Old Petrographic Microscope. Geology.

[31] Hemstapat R., Duangiad P., Tangketsarawan B., Phuagpan T., Chienwiwattanawong S., Tangsrianugul N., Ojida A., Wongkongkatep J. (2023). Improved Polarized Light Microscopic Detection of Gouty Crystals via Dissolution with Formalin and Ethylenediamine Tetraacetic Acid. Sci. Rep..

[32] Monosodium Urate Crystals. Rheumatology and Immunology Therapy.

